# The Relative Effects of Economic Growth, Environmental Pollution and Non-Communicable Diseases on Health Expenditures in European Union Countries

**DOI:** 10.3390/ijerph16245115

**Published:** 2019-12-14

**Authors:** Daniel Badulescu, Ramona Simut, Alina Badulescu, Andrei-Vlad Badulescu

**Affiliations:** 1 Department of Economics and Business, Faculty of Economic Sciences, University of Oradea, 410087 Oradea, Romania; dbadulescu@uoradea.ro (D.B.); simut.ramona@yahoo.com (R.S.); abadulescu@uoradea.ro (A.B.); 2 Faculty of Medicine, Iuliu Hatieganu University of Medicine and Pharmacy, 400012 Cluj-Napoca, Romania

**Keywords:** health expenditures, economic growth, environmental pollution, non-communicable diseases, EU countries

## Abstract

National and global health policies are increasingly recognizing the key role of the environment in human health development, which is related to its economic and social determinants, such as income level, technical progress, education, quality of jobs, inequality, education or lifestyle. Research has shown that the increase of GDP (Gross Domestic Product) per capita can provide additional funds for health but also for environmental protection. However, often, economic growth is associated with the accelerated degradation of the environment, and this in turn will result in an exponential increase in harmful emissions and will implicitly determine the increasing occurrence of non-communicable diseases (NCDs), mainly cardiovascular diseases, cancers and respiratory diseases. In this paper, we investigate the role and effects of economic growth, environmental pollution and non-communicable diseases on health expenditures, for the case of EU (European Union) countries during 2000–2014. In order to investigate the long-term and the short-term relationship between them, we have employed the Panel Autoregressive Distributed Lag (ARDL) method. Using the Pedroni-Johansen cointegration methods, we found that the variables are cointegrated. The findings of this study show that economic growth is one of the most important factors influencing the health expenditures both in the long- and short-run in all the 28 EU countries. With regards to the influence of CO_2_ emissions on health expenditure, we have found a negative impact in the short-run and a positive impact on the long-run. We have also introduced an interaction between NCDs and environmental expenditure as independent variable, a product variable. Finally, we have found that in all the three estimated models, the variation in environmental expenditure produces changes in NCDs’ effect on health expenditure.

## 1. Introduction

The worldwide political priorities concerning health have only recently started to include environmental issues; for a long time, public health and environmental protection have been considered somewhat separate concerns and budget chapters in the activities of governments and local communities [1,2]. However, communities and governments have gradually become aware of the importance of understanding the major impact of environmental degradation on human health, as well as the necessity of accurately estimating health costs associated with the quality of the environment. Improving the factors linked to the overall health status of the population (i.e., income levels, better working conditions, GDP (Gross Domestic Product) growth and health expenditure per capita, social status, lifestyle, diet, health education) has led to a considerable increase in life expectancy, not only in the more developed economies and regions, but also in most developing countries of the world. Concurrently, however, data show that the negative impact of pollution on public health, also due to human activities, has increased [1,3].

In this context, our paper aims at examining how health expenditures are influenced by the environmental pollution and non-communicable diseases (NCDs) in the case of EU countries, by using a panel data analysis. The article is organized as follows: in the following section, we provide a quantification of the disease burden and health costs due to environmental degradation and NCDs; in the third section, we review the literature on the influence of air pollution and use of renewable energies on health expenditure; in the fourth section, we present the methodology and sources of data; in the fifth section, we provide the results; in the seventh section we discuss the results; finally, we conclude and present the implications of the study but also the limitations. In the Appendix A, we present the statistical processing and results.

## 2. Quantification of the Disease Burden Attributable to Environmental Risk Factors

The relationship between the incidence of a significant number of diseases and the quality of the environment represents a long-term concern for international organizations; mainly the World Health Organization (WHO), but also Organisation for Economic Co-operation and Development (OECD) or EU have manifested concerns and interest for this topic. Thus, the WHO has regularly commissioned in-depth analyses regarding the impact of environmental factors upon health in the case of the citizens of their member states [4,5,6,7], in order to enable policy-makers from health and environment-related sectors to better set priorities for preventive actions (see Table 1). In this respect, studies and researches have been developed to better understand and respond to the environmental burden of diseases attributable to three selected risk factors, i.e., unsafe water, sanitation and hygiene; indoor air pollution from solid fuel use; and outdoor air pollution [6]. WHO estimates show that environmental risk factors contribute up to 22% to the global burden of disease from all causes (in DALYs—Disease-adjusted life year), and up to 23% to all deaths [7].

### 2.1. Environment and Non-Communicable Diseases (NCDs)

In 2016, air pollution was the second largest risk factor of non-communicable diseases (NCDs) worldwide, with 91% of the world population being exposed to harmful pollution levels in ambient air [9,10,11]. Indoor air pollution, especially given the inefficient and dangerous heating and cooking technologies, are currently affecting over 40% of the world population, especially in low and middle level income countries. It is estimated that environmental and household air pollution is responsible for 24% of cases of stroke, 25% of ischemic heart disease, 28% of lung cancer and 43% of chronic obstructive respiratory disease [12]. Various toxic chemicals and combinations thereof account for over 3 million deaths from NCDs in 2016, mainly from cardiovascular disease, chronic obstructive pulmonary disease and cancers. These include neurological and psychological disorders [7,13], with occupational carcinogens and airborne exposure to chemicals having caused 882,000 deaths in 2016 [14]. During the last decade, phenomena such as population growth and aging have led to an increase in the proportion of population vulnerable to NCDs determinants, especially due to air pollution and lack of physical activity (predominantly in urban agglomerations). Last but not least, an important issue is that the worsening of climate change has already begun to increase cardiovascular and respiratory mortality due to more frequent episodes of heat-waves [3,15].

Unfortunately, the future will be even more challenging, with demographic changes, urbanization, world economic growth rates and higher living standards generating significant environmental consequences. If current growth patterns and poorly performing energy policies will continue, the share of fossil fuel-based energy in the global energy mix will remain at around 85% [16]. This in turn translates into the following: during the 2050′s time horizon, a substantial increase in SO_2_ and NOx emissions in key emerging economies is foreseen to occur, and this will lead and contribute to an increase in premature deaths linked to urban air pollution (particulate matter and ground-level ozone), and a high burden of diseases stemming from exposure to hazardous chemicals, particularly in developing and poor countries (see Table 2). According to OECD forecasts, “air pollution is set to become the world’s top environmental cause of premature mortality” [16] (p. 24); by 2050, the number of premature deaths from air pollution is expected to reach 3.6 million individuals per year globally (more than double than the 2010 levels), with most deaths occurring in BRICS (Brazil, Russia, India, China and South Africa) countries [16], despite of increasing investments in health care (especially in Russia) and impressive growth in the consumption of medical services (in China) in recent decades [17,18,19]. Even high developed countries that will manage to decrease the CO_2_, NOx and carbon black emission levels are still at risk of having a significant weight attributed to the mortality rates caused by the previously mentioned factors, as they face an ageing population concentrated in cities with relatively high pollution. Finally, the burden of diseases related to exposure to hazardous chemicals is significant worldwide, but most severe in developing countries, where chemical safety measures are still insufficient, and investments in environmental protection are being overshadowed by the prospects of faster and accelerated economic growth.

For European countries, despite the considerable efforts that have been made to improve the quality of the environment and to reduce the effects of pollution on human health, the impact of these factors is still quite significant, with national health and environmental budgets shrinking and being burdened. Thus, the WHO reports that factors such as access to clean water and hygienic sanitation services, housing conditions, air quality, work environment and exposure to extreme weather conditions continue to be responsible for 13–20% of the burden of diseases across Europe. Moreover, evidence suggests that air pollution accounts for eight months on average (and more than two years in the most polluted cities) of lost life [20], and many of these polluted cities are located in Europe.

### 2.2. Health Costs Related to Environmental Degradation

The impact of environmental degradation on human health does not refer only to its influence upon the quality of life, but also on health care spending, productivity and income losses. Thus, both the direct and indirect costs of the impact of environmental degradation on human health can be deduced, and the estimates of these costs show that measures aiming at improving the quality of the environment can be very valuable investments for the health and prosperity of both individuals and society.

Direct healthcare costs for environmental diseases can be estimated by using the environmental burden of diseases and health expenditure data. According to OECD data [3], direct health care costs due to environmental degradation are substantial, of up to 0.5% of the world’s GDP. Both the share of health expenditure in GDP, and the environment-related share of the burden of disease considerably vary from one country to another.

Overall, the estimate of the share of environment-related human health loss is as high as 5% for high income OECD countries, 8% for average income OECD countries and 13% for non-OECD countries [3]. Irrespective of the type of costs (direct and indirect) or the calculation method, the loss of health due to environmental degradation is substantial and requires consistent interventions. These environmental interventions can, in turn, save money on health care expenditures.

In 2013, premature deaths due to major NCDs (i.e., cardiovascular diseases, cancers, respiratory diseases and diabetes) cost the EU economies 0.8% of GDP [21]. EU-specific phenomena such as population ageing and the rapid implementation of new diagnostic and therapeutic technologies contribute to a steady increase in the share of health expenditure in GDP, but especially in high- and middle-income countries, NCDs are responsible for the largest part of these healthcare costs [21,22].

## 3. Literature Review

The links between the quality of the environment and the population health, the incidence of illnesses and deaths and their burden on public health expenditure are scientifically and practically proven facts. Researchers and policymakers, however, need to understand the reasons behind the occurrence of these situations and what actions are to be taken in healthcare and health related spending. Thus, by carefully calibrating these measures, policymakers can either resize these expenditures or maximize the effect of the funds available for health-care, and subsequently, for the environmental protection.

According to The European Health Report 2012, the social and environmental determinants of healthcare comprise a full set of social and physical conditions in which people live and work, including socioeconomic (i.e., income level and security, employment, gender and years of education), demographic, environmental and cultural factors, together with the healthcare system [20]. Alongside income (or GDP/capita), which is considered the most important factor explaining differences across countries regarding the level and growth of total healthcare expenditures [23,24,25,26,27], scholars and practitioners include population age structure and epidemiological needs [28,29], technological progress and variation in medical practice [30] and health system characteristics, through service provision [31], financing structure [26] and external funds (especially in developing countries) [29,32,33,34]. Income (at national and individual level) is, however, not the only factor associated with health-care spending, and data show notable differences regarding health-care spending between countries with similar income levels. Moreover, Hansen and King [35] considered that income levels could not be sufficient enough to assess health-care expenditure, and Blomqvist and Carter [36] demonstrated that health expenditure could increase faster than economic growth rates.

In different words, the socio-economic viewpoint is far from being complete, and many researchers consider that, in the last century, socio-economic determinants had excessively shaped the philosophy and actions undertaken in the field of public health; meanwhile, the environment and natural systems have been considered as implicit support and a (practically inexhaustible) resource for human development [2], and “somehow, modern public health had almost forgotten the primacy of the human environmental interface, despite this being a component part of the original sanitarian vision” [37]. Therefore, many researchers and international decision-making organizations consider that environmental determinants should be included in the public health equation.

Despite a plethora of literature concerning the relationship between health expenditure and economic growth (estimated by GDP/capita), there are relatively few studies addressing the influence of multiple determinants (such as economic growth, renewable energy consumption and the environmental protection expenses) on health expenditure. Thus, in the following paragraphs, we will firstly review the studies addressing the impact of environmental pollution (air, in particular, as well as water and soil), and afterwards, those related to the issue of renewable energy.

### 3.1. Health Expenditure, Economic Growth and Air Pollution

Jerrett et al. [38] explored the relationship between health care expenditures and environmental factors in Canada, and found that both the total toxic pollutant emissions and per capita environmental expenditures are in a significant relationship with health expenditure. They argue that higher pollution regions/countries report higher per capita health expenditure, while environmental investments and responsible environmental behavior lead to lower healthcare spending. In the case of developed economies (i.e., Australia, by analyzing the relationship environmental quality, or degradation, and health expenditure) over the 1995–2017 time period, Moosa [39] arrived at a rather unusual (counter-intuitive) conclusion: when a country is on a downward Environmental Kuznets Curve (EKC), healthcare expenditure is negatively related to environmental degradation. Apergis et al. [40] found a positive impact of CO_2_ emissions on health-care spending in 50 U.S. states and this effect is stronger for those states which allocate higher funds for health-care expenditure. Blázquez-Fernández et al. [41] analyzed the impact of per capita income and environmental air quality variables on health expenditure determinants in 29 OECD countries, during the 1995–2014 time period. Their results show that per capita income has a positive effect on health expenditure, but this relationship is not as statistically significant as expected when lag-time is incorporated [41] (p. 389). The authors try to answer the question regarding how air pollution could affect health care expenditure by including in the relationship between health expenditures and income (per capita) several other air-pollutants, such as nitrogen oxides, sulfur oxide and carbon monoxide emissions. Their conclusions show clear health related benefits, and implicitly, savings on health care expenditure, when decision-makers support and promote growth based on cleaner fuels and pay more attention to the quality of the environment. However, different levels of economic development, budgetary restrictions and constraints, various fiscal policy frameworks, or health policies result in different outcomes and results in each country.

Other researches show that the effects of environmental degradation and, implicitly, air pollution are more severe in developing countries [42], making it more complicated to counter the negative effects and improve healthcare. Preker et al. [43] estimated the total tangible healthcare expenditure attributable to human pollution affecting air, soil and water. The conclusions, although somewhat expected, are alarming in terms of the negative impact of pollution, especially for fragile healthcare systems in poor countries. Thus, for 2013, the authors found that healthcare expenditure attributable to man-made pollution accounts between 3% and 9% of global spending on health care. Although the expenditure levels of developing countries hold a share of below 15% of this total, “the relative share of spending for pollution related illness is substantial, especially in very low-income countries. Cancer, chronic respiratory and cardio/cerebrovascular illnesses account for the largest health care spending items linked to pollution even in lower- and middle-income countries” [43] (p. 711). The authors pointed out that when adding to the expenditure list the intangible losses and the opportunity costs generated by lower labor productivity due to polluted environment, the financial impact of pollution on health becomes quite substantial, both at individual (or household) level and national level, and especially among the poorly developed countries [44].

By analyzing Sub-Saharan African Countries, Zaman et al. [45] found out that although increasing consumption of fossil fuel energy is associated with GDP growth, and implicitly the possibility of additional health allocations and increased life expectancy, it is also associated with carbon dioxide emissions, which increase spending on health per capita. On one hand, increases in life expectancy significantly decrease the health care provisions of per capita health levels; on the other hand, life expectancy is negatively affected by poor sanitation and low environmental protection spending levels, which also adds to the budgets dedicated to healthcare expenditures.

The case of China, from this point of view at least, is paradigmatic. Thus, many researchers have noted that China’s rapid economic growth has been accompanied by a considerable increase in pollution, which has caused extensive damage to the health of its inhabitants, and has implicitly altered public health spending; in the same time, it has also increased inequalities. Hence, Yang and Liu [46] have shown that the deterioration of health caused by pollution further increases the inequality of access to healthcare services, affects the level of disposable income and its share budgeted for healthcare. The research findings highlight that the negative effects are particularly acute for people with low incomes coming from poor rural areas, thus validating, to some extent at least, the concept of an “environment-health-poverty trap”. Lu et al. [47], by investigating 30 Chinese provinces between 2002–2014, or Hao et al. [48], by using panel data on Chinese provinces for 1998–2015 time period, proved the negative effect of environmental pollution on public health, increased medical expenses of Chinese residents, but also that public services and education can contribute to the decrease of the individual burden of medical expenses.

Using a model examining the relationship between health expenditure, income, CO_2_ emissions and PM_10_ (particulate matter) emissions, for a panel of MENA (Middle East and North Africa) countries during 1995–2014, Khoshnevis Yazdi and Khanalizadeh [49] found an “overwhelming evidence between health expenditures and its determinants” [49] (p. 1189). Thus, economic growth in these countries will increase the consumption of fossil fuels, environmental degradation, and will lead to higher risk of pollution-induced health diseases and mortality. They will lead to an increase in healthcare expenditures as share of the GDP, but in the context of significant budget constraints, this means diminishing funds allocated towards other sectors (such as education or environmental protection). However, without adequate education and with poor environmental protection, the negative health effects will worsen and the vicious circle will continue. Even so, the authors state that the only solution is to raise real GDP in MENA countries, in order to create resources available for investments in key sectors (including the health sector).

Ghorashi and Alavi Rad [50] examined the causal relationship between CO_2_ emissions, health expenditures and economic growth, by using dynamic simultaneous equation models for Iran over the 1972–2012 time period. Their results confirm previous studies [51,52,53] regarding the bidirectional causality relationship between CO_2_ emissions and economic growth, and, respectively, the unidirectional causality relationship between health expenditures and economic growth. The authors show that without an active environmental protection policy and an increase of technological transfer pace, the environmental damage can severely affect both the health levels of the population, but it can also compromise economic development. A similar solution, of “controlling pollution, particularly CO_2_ emissions and health expenditures without compromising economic growth” has been proposed also for the case of Pakistan by Wang et al. [54], who found a bidirectional relationship between health expenditures and CO_2_ emissions, and between health expenditure and economic growth in the long run, as well as a unidirectional causality from carbon emissions to health-related expenditures in the short-run.

### 3.2. Health Expenditure, Economic Growth and Renewable Energy

Although relatively newly addressed, the relationship between renewable energy and health expenditure can provide valuable insights for better understand the nexus health expenditure—GDP environment. Romm and Ervin [55] considered that “the vast majority of air pollution is energy related”; as the world’s states develop, they require increased amounts of energy, which is typically produced from traditional resources and by using pollution-intensive methods. Thus, we note that “environmental problems at an urban, regional, and global levels will be seriously aggravated, at a terrible cost to human health and quality of life” [55] (p. 398). For these authors, the key of sustainable development is through pollution prevention and resource-efficient technologies, in order to improve the environment, while at the same time lowering the energy bills of consumers and businesses. Without adhering to a certain type of economic intervention, they admit that this option requires large levels of public (and private) investments, enforcement measures and sanctions; however, the alternatives are simple: we either have a “sustainable, profitable, and environmentally sound” development, or a “on short term, costly, and potentially devastating from an environmental and human health perspective” [55] (p. 398) type of development. Higher costs related to the implementation and support of renewable energy can be recovered, however, from the health-related savings associated with the effects of pollution. In different words, investments in sustainable development are “the nexus of energy, transportation, air quality, climate change and health” [56] (p. 48); these investment will lead to a reduction in carbon emissions, which will significantly improve the health problems associated with climate change and air pollution, and said investments will implicitly reduce the burden of health costs associated to the quality of the environment. In fact, the environmental risk factors identified as responsible for the majority of disease burdens and for the excessive overloading of health-care spending can largely be avoided by implementing effective and sustainable policy interventions: improving air quality, access to safe drinking and sewage, sanitation and clean energy sources and by significantly contributing “to the achievement of the Millennium Development Goals of environmental sustainability, health and development” [57] (p. 2182).

Ben Jebli [58] investigated the relationship between health, real GDP, combustible renewables and waste consumption, rail transportation and carbon dioxide (CO_2_) emissions for the case of Tunisia for the 1990–2011 period. He found that, in the short-run, there is a unidirectional causality ranging from real GDP to health, from health-care to combustible renewables and waste consumption and from all variables to CO_2_ emissions. In the long run, combustible renewables and waste consumption have a positive and statistically significant impact on health levels, while CO_2_ emissions and rail transportation both contribute to the decrease of the health indicator. Similar to Romm and Ervin [55] or Erickson and Jennings [56], Ben Jebli [58] proposes to decision-makers to exploit waste and renewable fuels, to use renewable energy for national rail transportation, to invest in renewable energy projects in order to eliminate pollution caused by emissions, thus ensuring the economy’s growth and to reduce energy dependence, and subsequently, to improve health quality.

In a study aimed at investigating the long run relationship between health expenditure, real income, CO_2_ emissions and renewable energy consumption (all per capita) for BRICS-T countries (Brazil, Russia, India, China, South Africa, Turkey) during 2000–2015, Çetin [59] confirmed that when “CO_2_ emissions per capita increases, health expenditure per capita will also increase due to the worsened health status caused by the pollution effect” [59] (p. 365). Moreover, he showed that “renewable energy sources not only support sustainable development but they also mitigate health costs and increase the savings levels even the impact of CO_2_ emissions on health expenditure is still stronger than the remedial impact of renewable energy sources” [59] (pp. 365–366), i.e., the renewable energy sources could temper health expenses for environmental attributable diseases, but not decisively. In a study conducted on a panel of ASEAN (Association of South East Asian Nations) member states, Khan [60] confirmed that polluting industrial activities and CO_2_ emissions “have both a significant and negative effect on human health and environmental sustainability and increase the public health expenditure” [60]; meanwhile, the use of renewable energy will assist in the increase of economic performance and implicitly, aid in the efforts to increase environmental protection.

## 4. Data and Methodology

In this article, we aim at examining the long-term relationship between health expenditure (HE) and other determinants, such as: Gross Domestic Product per capita (GDP), emissions of carbon dioxide (CO_2_), environmental expenditure (ENV), renewable energy consumption (RENEW) and deaths caused by non-communicable diseases (NCDs) in the 28 European Union countries. For the purpose of this study we consider the following series as proxies of non-communicable diseases: (1) the diseases of the respiratory system (RESP), (2) the diseases of the circulatory system (CARDIO) and (3) the malignant neoplasms (CANCER), which are considered (alongside diabetes) as major NCDs in the EU [21]. Regarding the selection of variables, some considerations are necessary: we have selected all deaths caused by NCDs, i.e., RESP, CARDIO and CANCER, although some cardiac diseases and many respiratory diseases are sustained through infectious (aggravated or not by the presence of the environmental risk factors or changes in climate [7] and [61]), given that the main database we used does not discriminate more in-depth [62]. Moreover, risks factors for non-communicable diseases also come from other areas, but are related to the environment, i.e., overweight, low physical activity and unintentional and intentional injuries [7]. For example, at a global level, environmental factors accounted for 19% of the injuries from interpersonal violence [7]. However, given the above-mentioned statistical reasons, the deaths due to unintentional injuries (i.e., road traffic accidents, unintentional poisonings, falls, fires, drownings) or intentional injuries (i.e., self-harm or interpersonal violence), although related to environmental risk factors or country income level, have not been considered for the purposes of the present analysis.

In order to evaluate the relationship between these variables, we have used a panel data analysis. The panel data were retrieved from the earliest possible year, namely the year 2000, and continued until the last available year, namely 2014, and a balanced panel of these countries was constructed. Table 3 provides information about variables and the sources of data. Since all variables are expressed in different units of measurement (i.e., current prices, current international $, metric tons, terajoule), before analyzing any aggregation, the data needed to be transformed into a normal form. Thus, we converted the variables into their natural logarithmic forms, as the natural logarithms of all variables smooth out the entire data used for analysis. Additionally, if the coefficients are estimated by variables that have been transformed into their natural logarithmic forms, the results can be interpreted in terms of elasticity.

Time series variables have different properties, such as those related to stationarity. The testing of stationarity can be performed by unit root tests, which can be determined on the basis of a presumption of cross section independence. For panel data, the variables of the analyzed countries are correlated with each other due to regional interconnections between these countries. Thus, in order to test for cross section dependence, we used the Lagrange multiplier (LM) test of Breusch and Pagan [65], and the cross-sectional dependence (CD) test suggested by Pesaran [66]. The equation of the LM test according to Breusch and Pagan is:

(1) $$LM=\sqrt{\frac{1}{N(N-1)}}\sum_{i=1}^{N-1}\sum_{j=i+1}^{N}\left(T\rho_{ij}^{2}-1\right)$$

These tests have as null hypotheses the cross-sectional independence and the alternative hypotheses is cross-sectional dependence. Given the shortcomings of the Breusch and Pagan LM test when N is high, Pesaran [66] proposed an alternative based on pairwise correlation coefficients rather than their squares used in the LM test. Thus, the equation of the CD test is:

(2) $$CD=\sqrt{\frac{2T}{N(N-1)}}\left(\sum_{i=1}^{N-1}\sum_{j=i+1}^{N}\left(\rho_{ij}\right)\right)$$

where N denotes the sample size, T denotes the time period of the study and $\rho_{ij}$ denotes the correlations’ coefficients of the residuals of different cross-sections of country i and j.

In Table A1, we present the results of cross-sectional dependence by using the LM test suggested by Breusch and Pagan [65] and CD test suggested by Pesaran [66]. The results demonstrate that the null hypotheses regarding the cross-sectional independence is rejected and the alternative hypotheses regarding cross-sectional dependence is accepted at the significant level of 1%.

After the confirmation of the cross-sectional dependence, the next step of the empirical analysis is to determine whether the series are stationary. In this regard, we employed three different types of panel unit root tests: Im, Pesaran and Shin W-stat [67], ADF—Fisher Chi-square [68] and PP—Fisher Chi-square [69]. It is important to note that all the series have the same order of integration. Thus, if the series are not stationary at the same level, cointegration analysis cannot be used. In this regard, if some series are stationary at level and others are stationary on first difference level, the ARDL (Autoregressive Distributed Lag) Bounds test will be used. It allows the use of data with different levels of stationarity, but limited at level I (0) and I (1) or a mixture of both and it can also estimate the cointegration equation with a very small number of sample cases (see Pesaran and Smith [70], Pesaran et al. [71,72]).

Table A2 shows the results of unit root tests, indicating that all variables are stationary (at a significance level of 5%) at first difference with intercept, except the variable Diseases of the respiratory system (RESP), which is stationary at its level, at a significance level of 5%. Therefore, we can state that our data suffers from either I (0) or I (1), and in addition, these estimations give us the possibility of estimating the short and long-run relationships along with the error correction coefficient. Moreover, as variables are static at level or on their first differences levels, applying cointegration analysis on the variables is possible.

In order to study the cointegration, the Pedroni Johansen cointegration and the Fisher test were performed. The results are presented in Table A3. We estimated the Pedroni [73] heterogenous panel cointegration test and used the panel fixed effect estimators [74]. The panel tests proposed by Pedroni [73] are based on the within-dimension approach, while the group tests are based on the between-dimension approach. The first includes four statistics: panel v-Statistic, panel rho-Statistic, panel PP-Statistic and panel ADF-statistics, while the group tests include three statistics: group rho-Statistic, group PP-Statistic and group ADF-statistics. All seven statistics are based on an average of the individual autoregressive coefficient related to the residual unit root tests of each country belonging to the panel data set. Considering the first panel cointegration tests, for two out of four Pedroni tests (i.e., panel PP-statistic and panel ADF-statistic), the null hypothesis suggests that there is a cointegration between the variables. Thus, we can state that there is a long-run relationship between health expenditure, GDP, CO_2_ emissions, renewable energy and non-communicable diseases. Similarly, for the group test based on between dimensions, the null hypothesis suggests that the variables are cointegrated for two out of three tests (i.e., group PP-Statistic and group ADF-statistics). According to previous literature, the results of the PP group statistics, which are both heterogeneous and non-parametric, are considered as having the highest power in the Pedroni test. In our case, the statistics of this group PP reject the null hypothesis due to a lack of cointegration at a certain level of significance, and in conclusion, the variables are cointegrated.

To estimate the long- and short-term relationship between studied variables, and also in order to investigate the possible heterogeneous dynamic problem in different countries, the most appropriate method that can be used for the analysis of dynamic panels is the ARDL (p, q) model in the error correction form. The model estimation will be based on the Pooled mean group (PMG) developed by Pesaran et al. [71]. The general ARDL specification is formulated as follows:

(3) $$Y_{it}=\sum_{j=1}^{p}\alpha_{ij}y_{i,t-j}+\sum_{j=0}^{q}\beta_{ij}X_{i,t-j}+\mu_{i}+\varepsilon_{it}$$

where i represents the number of cross sections (i = 1, 2, …, N), t represents the time (t = 1, 2, …, T), $X_{i,t-j}$ is a vector of K × 1 explanatory variables for group i and $\mu_{i}$ represents the fixed effect.

A specific feature of co-integrated variables is their response to any deviation from long-term equilibrium. This feature is based on the error correction dynamics of the variables in the system that are affected by the deviation from equilibrium. Thus, this equation can be re-parametrized [75] into the error correction (ECM) equation:

(4) $$\Delta y_{it}=\delta_{i}\left(y_{i,t-1}-\beta_{i}X_{i,t}\right)+\sum_{j=1}^{p-1}\gamma_{i,j}^{}\Delta y_{i,t-j}+\sum_{j=0}^{q-1}\lambda_{i,j}^{}\Delta X_{i,t-j}+\mu_{i}+\varepsilon_{it}$$

where y is the natural logarithm of health expenditure per capita, X is a set of independent variables including the natural logarithm of GDP, CO_2_, renewable energy, environmental expenditure and non-communicable diseases and $\gamma_{i,j}$ and $\lambda_{i,j}$ represent the short-run coefficients of exogenous and endogenous variables respectively. $\delta_{i}$ is the error correction parameters that measure the speed of adjustment and $\beta_{i}$ is the long-run parameters that captures the long run equilibrium relationship between y and X, with short run effects measured by $\lambda_{i,j}^{}$, which represent the parameters associated with the ΔX variables.

Therefore, the Pooled mean group allows the determination of the short-run and long-run coefficients, the intercepts and the speed of adjustment to the long run equilibrium relationship. The main requirements for the consistency of this technique reside, firstly, in the existence of a long-run relationship between the variables analyzed, and as this happens, the error correction parameter has to be negative and statistically significant. Moreover, the residual of the error correction model must be independent and the exogenous variables can be treated as independent. For these conditions, we include the lags of the ARDL (p, q) model for the endogenous variable (p) and exogenous variable (q) in the error-correction form. This estimator is useful especially when there are reasons to expect long-run equilibrium relationships between variables to be similar across the European Union countries, because they might have similar levels of economic growth, similar levels of health expenditure and similar degrees of pollution.

In terms of a short-run relationship and the slope coefficients between individual countries, they are allowed to be country-specific, due to the very comprehensive impact of external shocks and stabilization policies.

## 5. Results

The long-term elasticities are estimated by ARDL panel method for all the three models. All models presented in Table A4 take the same form as Equation (2), augmented to include each individual non-communicable disease: the malignant neoplasms (CANCER), the diseases of the circulatory system (CARDIO) and the diseases of the respiratory system (RESP). In order to detect how variation in environmental expenditure produces changes in NCD’s effect on health expenditure and how variation in NCDs produces changes in environmental expenditure’s effect on health, we have included a product-variable in each model, LnENV x lnNCDs.

Table A4 shows both long-run and short-run estimation results. With regards to the long-run relationship, the error correction term (ECT) involves a possible long-run relationship between the variables in all three models, because ECT is statistically significant at 1% level and is negative. In line with other studies [41,48,49,76,77,78,79,80], the GDP per capita results show a significant and positive long-run effect on health expenditure per capita in 28 EU countries in all three models. Thus, the 1% increase in GDP per capita could lead to an increase of 2.39%, 1.08% and 1.14%, respectively, of health expenditure in the long run. The high values of the coefficients have shown that, as expected, GDP is an important factor in EU countries in terms of the levels and increases of health expenditures in the long-run.

Regarding the effect of CO_2_ emissions per capita on health expenditure, the long-run estimations revealed that emissions cause an increase in health expenditure in all 28 EU countries, and in all three models. The CO_2_ emissions coefficient is statistically significant at the 1% level. In the selected sample, the CO_2_ emissions could lead to an increase of 0.7%, 0.6% and 1%, respectively, of health expenditure in the long-run. The implication is that air pollution generates increases in health expenditure, but at lower levels compared to the GDP. Therefore, if emerging economies would accept the price of worsening the environment by deploying an ample economic development, these economies should also accept to cover higher health expenditure due to the negative effects of air pollution.

When analyzing the impact of renewable energy on health expenditure, there is a 1% increase in renewable energy consumption per capita, which could lead to a decrease of 0.26%–0.30% of health expenditure per capita in the long-run. These results are consistent with the findings of Çetin [59] and Khan [60], and show that the use of renewable energy could reduce the possible effects of pollution on health. However, the long-run coefficients in the sample show that the increasing impact of CO_2_ emissions on health expenditure is higher than the low impact of renewable energies.

The coefficients of the product term in our equations do not have a simple causal interpretation, as they describe how the causal effect of an explanatory variable on the dependent variable is affected by the variation of the other variable [81]. Thus, in the interaction of non-communicable diseases and environmental expenditure on health-care, the product term’s coefficient seems to describe both how variation in environmental expenditure produces changes in NCD’s effect on health expenditure, and how variation in NCDs produces changes in environmental expenditure’s effect on health. Thus, in the first model that includes the diseases of the respiratory system (RESP), the product term coefficient is negative, while the coefficients of the variable ENV and RESP are positive. Consequently, a 1% increase in the diseases of the respiratory system (RESP) could lead to an increase of 2.13% of health expenditure when no environmental expenditure exists. However, variation in environmental expenditure produces changes in the diseases of the respiratory system (RESP) with an effect on health expenditure; thus, a 1% increase in the environmental expenditure leads to a decrease of 0.14% of changes in the diseases of the respiratory system (RESP) and their effect on health expenditure (from 2.13% to 1.99%). In the second model, which includes the diseases of the circulatory system (CARDIO), the results are similar: a 1% increase in the diseases of the circulatory system (CARDIO) could lead to an increase of 2.14% in regards to health expenditure when no environmental expenditure exists. However, when investigating how the variation in environmental expenditure produces changes in the effects of diseases of the circulatory system (CARDIO) on health expenditure, the results are as follows: a 1% increase in the environmental expenditure leads to a decrease of 0.18% in changes of the diseases effect on health expenditure (i.e., from 2.14% to 1.96%). The long-run coefficient of the malignant neoplasms (CANCER) is statistically significant at 5% level and this non-communicable disease could lead to an increase of 0.92% of health expenditure in the long-run. Resuming, when the quality of the environment decreases, there is a negative impact on human health, which will lead to a health deterioration, by spending more in order to better take care of one’s health.

## 6. Discussion

The results of the statistical analyses show that an increase of the GDP per capita is an essential element in supporting the increase of health-related spending. However, if this increase would be unsustainable (e.g., generating emissions of pollutants, chemicals, waste, energy from fossil fuels), it would not be able to cope with the negative effects of a polluted environment on population’s health. The incidence of NCDs associated with a polluted environment and exposure to hazardous chemicals (largely the result of industrial development, but also due to socio-economic inequalities) and increased environmental protection spending outweigh the positive effects of renewable energy production or eco-friendly goods.

For better understanding the direction of the relationship between health expenditures and GDP, CO_2_ emissions, renewable energy, environmental expenditure and the three analyzed non-communicable diseases, we have employed the panel causality tests developed by Dumitrescu and Hurlin [82]. This test can be used for the unbalanced and heterogeneous panels, even in the case of samples with very small T and N dimensions.

**Figure 1:**
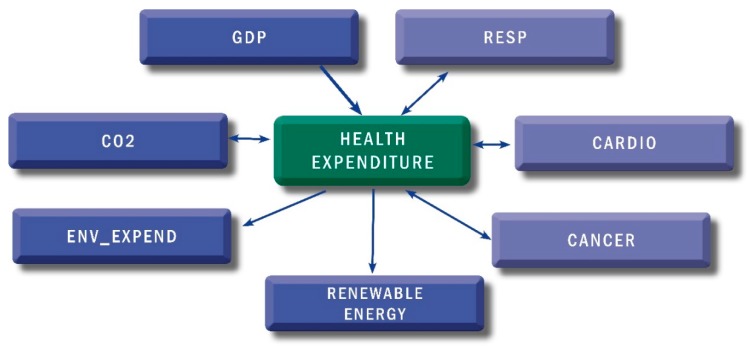
Dumitrescu-Hurlin panel causality test results. Source: Authors’ calculation using Eviews 9.

In Figure 1 we describe the results of the Pairwise Dumitrescu-Hurlin panel causality test. The test shows evidence of a unidirectional causal relationship ranging from GDP to health expenditure, health expenditure to renewable energy, and, respectively, from health expenditure to environmental expenditure, for the full sample. We found a causal bidirectional connection between health expenditure and CO_2_, respectively between health expenditure and the non-communicable diseases.

## 7. Conclusions

During the last few decades, we have witnessed a sustained endorsement of the primacy of socio-economic factors (i.e., economic power, distribution of knowledge and opportunities, education) in order to safeguard a proper human existence. Gradually, however, an increasingly rich scientific literature, accompanied by serious signals from international and non-governmental organizations, has highlighted the vital role of a functioning natural environment in sustaining human dignity, well-being and health [83].

In this article, we have analyzed both the long-run and the short-run relationship between economic growth, environmental pollution and NCDs on health expenditure in the 28 EU countries over the 2000–2014 time period, employing the Panel Autoregressive Distributed Lag (ARDL) method. Using the Pedroni Johansen cointegration, we have found that the variables are cointegrated. Our results show that the GDP has the greatest impact on health expenditure. Therefore, a 1% increase in GDP per capita could lead to an average increase of 2% of health expenditure in the long run, i.e., countries with higher GDP per capita have a greater potential for health expenditure growth. Regarding CO_2_ emissions, we found that they determine a decrease of health expenditure in the short-run and a growth in the long-run, i.e., a 1% increase in CO_2_ emissions per capita could lead to an increase between 0.6% and 1% of health expenditure in the long run (depending on which of the three deployed models is chosen). We can argue that the impact of CO_2_ emissions on health expenditure is dependent on economic growth, and this in turn would lead to higher health spending. Ideally, this growth should occur on the basis of clean, renewable energies (i.e., the mediating role of renewable energy), and the effects of this growth should generate increases of health expenditure. However, this may not apply for all countries, some EU member states included. Renewable energies influence the level of health expenditure in all EU countries, but to a lower degree than CO_2_ emissions. The long-run coefficients of our sample show that the increasing impact of CO_2_ emissions on health expenditure is higher than the (low) impact of renewable energies. Therefore, we consider that EU countries should carefully follow a development agenda of renewable energy sources, not simply as a means to protect the environment, but also to lower the costs of diseases attributable to environmental risk factors or the environmental protection expenditures. As expected, environmental expenditure also has a significant and positive impact on health expenditure. Moreover, the variation in environmental expenditure produces changes in NCD’s effect on health expenditure, i.e., a 1% increase in the environmental expenditure leads to a decrease in the NCDs effect on health expenditure. Therefore, when no environmental expenditure exists, a 1% increase in the diseases of the respiratory system (RESP) could lead to an increase of 2.13% of health expenditure. In case of the circulatory system (CARDIO) and malignant neoplasms (CANCER), in the long-term, a 1% increase of them could lead to an increase of 2.14% 0.92%, respectively of health expenditure when no environmental expenditure exists.

We have found that the relationship between the environment and health goes beyond the effects of economic development, pollution and NCDs, which nonetheless remain important aspects of public health. The results of our research can provide substantial arguments for political decisions in order to maximize the efficiency of healthcare spending concurrent with a better environmental protection, thus, enabling the increase of GDP both in the medium- and long-run, based on technological developments aiming at improving the quality of life, rather than targeting economic growth at all costs. Major political decisions which have consequences for society and negatively affect people’s health must not ignore the health-environment nexus. Health and environmental policies must be planned out for the medium and long-term, being effective and with positive consequences on human health, in order “to recognize and respond to the centrality of the environment for health” [2] (p. 106). By reinterpreting the essential consequences of the natural environment on human health, the environment should be recognized as a crucial element, of equal importance as socio-economic factors, on national and global policies for public health and for environmental protection.

In this study, we have faced some limitations, mainly related to the difficulty of obtaining a comprehensive dataset for a long period of time and a significant number of different variables. Thus, the time period taken into account was reduced to 15 years. Moreover, for some key variables, such as those related to non-communicable diseases morbidity, and which could have been included as explanatory variables in the estimated models, insufficient data were available for the entire analyzed period. These limitations, although they do not discredit the results of this analysis, should be addressed in subsequent studies. Furthermore, further research aiming at investigating all EU countries could include more variables related to environmental quality, variables related to different types of health expenditure and variables revealing the economic differences between developed and developing countries.

## Figures and Tables

**Table 1:** Percentage of deaths and Disease-adjusted life years (DALYs) attributable to five environmental risks (and to all five risks combined) by region, 2009.

Environmental Risk	World	Low- and Middle-Income Countries	High Income Countries
Percentage of deaths			
Indoor smoke from solid fuels	3.3	3.9	0
Unsafe water, sanitation, hygiene	3.2	3.8	0.1
Urban outdoor air pollution	2	1.9	2.5
Global climate change	0.2	0.3	0
Lead exposure	0.2	0.3	0
All five risks	8.7	9.6	2.6
Percentage of DALYs			
Indoor smoke from solid fuels	2.7	2.9	0
Unsafe water, sanitation, hygiene	4.2	4.6	0.3
Urban outdoor air pollution	0.6	0.6	0.8
Global climate change	0.4	0.4	0
Lead exposure	0.6	0.6	0.1
All five risks	8	8.6	1.2

Source: WHO, Global health risks: mortality and burden of disease attributable to selected major risks, 2009b [6]; WHO, Quantification of the disease burden attributable to environmental risk factors. WHO: Geneva, Switzerland, 2009c [8].

**Table 2:** Overview of premature deaths from selected environmental risks, forecast for 2010–2050 time period (in millions of people).

Year	Selected Environmental Risks				
	Particulate Matter	Ground-Level Ozone	Unsafe Water Supply and Sanitation *	Indoor Air Pollution	Malaria
2010	1.4	0.4	1.8	2.2	0.9
2030	2.3	0.6	1.1	2.2	0.7
2050	3.6	0.8	0.5	1.9	0.4

Source: OECD (Organization for Economic Co-operation and Development). OECD Environmental Outlook to 2050: The Consequences of Inaction. OECD Publishing: Paris, Brussels, 2012 [16]; Prüss-Ustün, A.; Wolf, J.; Corvalán, C.; Bos, R.; Neira, M. (WHO). Preventing disease through healthy environments: a global assessment of the burden of disease from environmental risks. WHO: Geneva, Switzerland, 2016 [7]. * Note: Child mortality only.

**Table 3:** Variables and Data Sources.

Variable Name	Description	Source
**Dependent Variable (Natural Logarithm)**		
lnHE	Health expenditure per capita (PPP, current international $)	World Bank [63]
**Independent Variables (Natural Logarithm)**		
lnGDP	GDP per capita (Current prices, euro per capita)	Eurostat [64]
lnCO_2_	CO_2_ emission per capita (metric tons per capita)	World Bank [63]
lnENV	Environment expenditure (million euro)	Eurostat [64]
lnRENEWABLE	Renewable energy consumption per capita (Terajoule)	Eurostat [64]
**Non-communicable diseases**		
lnRESP	Diseases of the respiratory system (number)	Eurostat [64]
lnCARDIO	Diseases of the circulatory system (number)	Eurostat [64]
lnCANCER	Malignant neoplasms (number)	Eurostat [64]

## Appendix A

**Table A1:** Cross-Sectional Dependence Analysis.

Variable	Breusch-Pagan LM Test		Pesaran CD Test	
	Statistic	Prob	Statistic	Prob
lnHE	5284.54	0.00	72.63	0.00
lnGDP	4415.94	0.00	64.62	0.00
lnCO_2_	2815.52	0.00	38.54	0.00
lnRENEWABLE	4425.52	0.00	66.09	0.00
lnENV	4879.30	0.00	69.70	0.00
lnRESP	1116.75	0.00	5.95	0.00
lnCARDIO	2773.90	0.00	37.96	0.00
lnCANCER	2925.75	0.00	38.64	0.00

Note: LM—Lagrange multiplier. CD—cross-sectional dependence. Probability (Prob.). Source: Authors’ calculation using Eviews 9.

**Table A2:** Panel unit root test.

Variable	Im, Pesaran and Shin W-Stat		ADF—Fisher Chi-Square		PP—Fisher Chi-Square	
	Level	First Difference	Level	First Difference	Level	First Difference
HE	−0.35	−7.60	55.10	156.32	43.00	159.64
	(0.36)	(0.00)	(0.50)	(0.00)	(0.89)	(0.00)
GDP	1.06	−6.791	49.77	143.65	51.37	133.72
	(0.85)	(0.00)	(0.70)	(0.00)	(0.65)	(0.00)
CO_2_	5.91	−13.95	25.10	267.22	29.30	295.59
	(0.99)	(0.00)	(0.99)	(0.00)	(0.99)	(0.00)
RENEWABLE	7.14	−10.66	17.00	213.57	9.98	233.73
	(0.99)	(0.00)	(0.99)	(0.00)	(0.99)	(0.00)
ENV	2.64	9.20	39.46	182.05	40.67	175.72
	(0.99)	(0.00)	(0.95)	(0.00)	(0.93)	(0.00)
RESP	−4.74		113.63		112.26	
	(0.00)		(0.00)		(0.00)	
CARDIO	0.86	−16.05	55.53	299.68	68.91	313.03
	(0.80)	(0.00)	(0.49)	(0.00)	(0.11)	(0.00)
CANCER	3.23	−13.72	40.65	263.94	38.34	310.49
	(0.99)	(0.00)	(0.93)	(0.00)	(0.96)	(0.00)

Note: ADF—augmented Dickey-Fuller, PP—Phillips-Perron. HE—health expenditure, GDP—Gross Domestic Product per capita, CO_2_—emissions of carbon dioxide, ENV—environmental expenditure, RENEW—renewable energy consumption, RESP—the diseases of the respiratory system, CARDIO—the diseases of the circulatory system, CANCER—the malignant neoplasms. Statistical probability values are given in parenthesis. Intercept without deterministic trend is the selected model for all unit root tests. Optimal lag length chosen by Akaike information criteria (AIC). Source: Authors’ calculation using Eviews 9.

**Table A3:** Pedroni Cointegration test results.

Statistics	Model 1—RESP		Model 2—CARDIO		Model 3—CANCER	
	Statistic	Prob.	Statistic	Prob.	Statistic	Prob.
Within-dimension						
Panel v-Statistic	−1.579124	0.9428	−1.865053	0.9689	−1.712348	0.9566
Panel rho-Statistic	4.915515	0.9998	4.406690	0.9997	4.213251	0.9996
Panel PP-Statistic	−1.872898	0.0305	−1.916072	0.0277	−1.961633	0.0249
Panel ADF-Statistic	−2.867907	0.0021	−4.509694	0.0000	−3.241310	0.0006
Between-dimension						
Group rho-Statistic	7.578138	1.0000	6.955135	1.0000	6.880314	1.0000
Group PP-Statistic	−4.979949	0.0000	−5.419722	0.0000	−5.364352	0.0000
Group ADF-Statistic	−3.792107	0.0001	−6.291378	0.0000	−4.575167	0.0000

Note: Optimal lag length chosen by Akaike information criteria (AIC). Probability (Prob.). Source: Authors’ calculation using Eviews 9.

**Table A4:** Panel Autoregressive Distributed Lag: Long-run and Short-run estimation.

Dependent Variable	Health Expenditure					
	Model 1 RESP		Model 2 CARDIO		Model 3 CANCER	
	Coefficient	Prob.	Coefficient	Prob.	Coefficient	Prob.
**Long Run**						
lnGDP	2.394454 ***	0.0000	1.082333 ***	0.0000	1.145709 ***	0.0000
lnCO_2_	0.762317 ***	0.0000	0.622213 ***	0.0021	1.005968 ***	0.0000
lnRENEWABLE			−0.262468 **	0.0124	−0.304537 ***	0.0001
lnENV	0.269130	0.3018	2.062498 ***	0.0026	−0.987343 **	0.0283
lnRESP	2.134922 ***	0.0000				
lnCARDIO			2.142316 **	0.0102		
lnCANCER					0.922279 **	0.0191
LnENV x lnRESP	−0.146130 ***	0.0000				
LnENV x lnCARDIO			−0.185771 ***	0.0064		
LnENV x lnCANCER					0.096141 **	0.0422
**Short Run**						
ECT	−0.263900 ***	0.0000	−0.282793 ***	0.0000	−0.272764 ***	0.0000
D(lnGDP)	0.319386 *	0.0533	0.359434 **	0.0146	0.357377 ***	0.0077
D(lnCO_2_)	−0.221160 **	0.0497	−0.054375	0.6926	−0.306034 ***	0.0062
D(lnRENEWABLE)			0.065164	0.4718	0.067259	0.4169
D(lnENV)	6.35497 **	0.0335	4.984683 ***	0.0000	−5.523548	0.7879
D(lnRESP)	7.934915 **	0.0379				
D(lnCARDIO)			5.329811 ***	0.0000		
D(lnCANCER)					−5.979762	0.7976
D(LnENV x lnRESP)	−0.68853 **	0.0332				
D(LnENV x lnCARDIO)			−0.452213 ***	0.0000		
D(LnENV x lnCANCER)					0.226611	0.9054
Intercept	−6.490450 ***	0.0000	−6.989633 ***	0.0000	−3.021624 ***	0.0000

*, **, *** denote rejection of the null hypothesis at the 0.10 level, 0.05 level and 0.01 level, respectively. Prob.—Probability. Source: Author’s calculation using Eviews 9.

## References

[1] OECD (2001). Human Health and the Environment. OECD Environmental Outlook 2001.

[2] Tait P., McMichael A., Hanna E. (2014). Determinants of health: The contribution of the natural environment. Aust. N. Z. J. Public Health.

[3] WHO Regional Office for Europe (2016). Urban Green Spaces and Health-A Review of the Evidence.

[4] WHO (2008). Environmental Burden of Disease Series.

[5] WHO (2009). Country Profiles of Environmental Burden of Disease.

[6] WHO (2009). Global Health Risks: Mortality and Burden of Disease Attributable to Selected Major Risks.

[7] Prüss-Ustün A., Wolf J., Corvalán C., Bos R., Neira M. (2016). Preventing Disease through Healthy Environments: A Global Assessment of the Burden of Disease from Environmental Risks.

[8] WHO (2009). Quantification of the Disease Burden Attributable to Environmental Risk Factors.

[9] Prüss-Ustün A., van Deventer E., Mudu P., Campbell-Lendrum D., Vickers C., Ivanov I., Forastiere F., Gumy S., Dora C., Adair-Rohani H. (2019). Environmental risks and non-communicable diseases. BMJ.

[10] WHO (2018). Ambient Outdoor Air Quality and Health. Fact Sheet.

[11] WHO (2018). Global Health Observatory-Data Repository. http://www.who.int/gho/database/en/.

[12] Landrigan P.J., Fuller R., Acosta N.J.R., Adeyi O., Arnold R., Basu N.N., Baldé A.B., Bertollini R., Bose-O’Reilly S., Boufford J.I. (2018). The Lancet Commission on pollution and health. Lancet.

[13] Arthur M., Institute for Health Metrics and Evaluation (2018). Global Health Data Exchange. http://ghdx.healthdata.org/gbd-results-tool.

[14] Wolf J., Prüss-Ustün A., Ivanov I., Mudgal S., Corvalán C., Bos R., Neira M. (2018). Preventing Disease through a Healthier and Safer Workplace.

[15] Kondo M., Fluehr J., McKeon T., Branas C. (2018). Urban Green Space and Its Impact on Human Health. Int. J. Environ. Res. Public Health.

[16] OECD (2012). OECD Environmental Outlook to 2050: The Consequences of Inaction.

[17] Jakovljevic M., Potapchik E., Popovich L., Barik D., Getzen T.E. (2017). Evolving health expenditure landscape of the BRICS nations and projections to 2025. Health Econ..

[18] Jakovljevic M., Fernandes P.O., Teixeira J.P., Rancic N., Timofeyev Y., Reshetnikov V. (2019). Underlying Differences in Health Spending Within the World Health Organisation Europe Region—Comparing EU15, EU Post-2004, CIS, EU Candidate, and CARINFONET Countries. Int. J. Environ. Res. Public Health.

[19] Preker A.S., Jakab M., Schneider M., Mossialos E., Dixon A., Figueras J., Kutzin J. (2002). Health financing reforms in central and eastern Europe and the former Soviet Union. Funding health care: Options for Europe.

[20] WHO Regional Office for Europe (2012). Social and Environmental Determinants of Health and Health Inequalities in Europe: Fact Sheet.

[21] European Commission The European Commission’s Science and Knowledge Service. Cost of Non-Communicable Diseases in the EU. https://ec.europa.eu/jrc/en/health-knowledge-gateway/societal-impacts/costs#_oecdec2016.

[22] OECD/EU (2018). Health at a Glance: Europe 2018: State of Health in the EU Cycle.

[23] Newhouse J. (1977). Medical-care expenditure: A cross-national survey. J. Hum. Resour..

[24] Getzen T. (2000). Health care is an individual necessity and a national luxury: Applying multilevel decision models to the analysis of health care expenditures. J. Health Econ..

[25] Musgrove P., Zeramdini R., Carrin G. (2002). Basic patterns in national health expenditure. B World Health Organ..

[26] Van der Gaag J., Stimac V. (2008). Towards a New Paradigm for Health Sector Development.

[27] Ke X., Saksena P., Holly A. (2011). The Determinants of Health Expenditure: A Country-Level Panel Data Analysis. Working Paper of the Results for Development Institute (R4D).

[28] Di Matteo L., Di Matteo R. (1998). Evidence on the determinants of Canadian provincial government health expenditures: 1965–1991. J. Health Econ..

[29] Lu C., Schneider M.T., Gubbins P., Leach-Kemon K., Jamison D., Murray C.J. (2010). Public financing of health in developing countries: A cross-national systematic analysis. Lancet.

[30] Newhouse J. (1992). Medical care costs: How much welfare loss?. J. Econ. Perspect..

[31] Gerdtham U., Jönsson B., MacFarlan M., Oxley H. (1998). The determinants of health expenditure in the OECD countries: A pooled data analysis. Health, the Medical Profession, and Regulation.

[32] Farag M., Nandakumar A.K., Wallack S.S., Gaumer G., Hodgkin D. (2009). Does funding from donors displace government spending for health in developing countries?. Health Affair..

[33] Nemec J., Kolisnichenko N. (2006). Market-based health care reforms in Central and Eastern Europe: Lessons after ten years of change. Int. Rev. Adm. Sci..

[34] Chang A.Y., Cowling K., Micah A.E., Chapin A., Chen C.S., Ikilezi G., Sadat N., Tsakalos G., Wu J., Zhao Y. (2019). Past, present, and future of global health financing: A review of development assistance, government, out-of-pocket, and other private spending on health for 195 countries, 1995–2050. Lancet.

[35] Hansen P., King A. (1996). The determinants of health care expenditure: A cointegration approach. J. Health Econ..

[36] Blomqvist Å., Carter R. (1997). Is health care really a luxury?. J. Health Econ..

[37] Lang T., Rayner G. (2012). Ecological public health: The 21st century’s big idea?. BMJ.

[38] Jerrett M., Eyles J., Dufournaud C., Birch S. (2003). Environmental influences on health care expenditures: An exploratory analysis from Ontario, Canada. J. Epidemiol. Community Health.

[39] Moosa N. (2019). CO_2_ Emissions, Environmental Degradation, and Healthcare Expenditure: Evidence from Australia. Manag. Econ. Res. J..

[40] Apergis N., Gupta R., Lau C., Mukherjee Z. (2018). US state-level carbon dioxide emissions: Does it affect health care expenditure?. Renew. Sust. Energy Rev..

[41] Blázquez-Fernández C., Cantarero-Prieto D., Pascual-Sáez M. (2019). On the nexus of air pollution and health expenditures: New empirical evidence. Gac. Sanit..

[42] Brinda E.M., Kowal P., Attermann J., Enemark U. (2015). Health service use, out-of-pocket payments and catastrophic health expenditure among older people in India: The WHO Study on global AGEing and adult health (SAGE). J. Epidemiol. Community Health.

[43] Preker A., Adeyi O.O., Lapetra M.G., Simon D.-C., Keuffel E. (2016). Health Care Expenditures Associated with Pollution: Exploratory Methods and Findings. Ann. Glob. Health.

[44] Jakovljevic M., Jakab M., Gerdtham U., McDaid D., Ogura S., Varavikova E., Merrick J., Adany R., Okunade A., Getzen T.E. (2019). Comparative financing analysis and political economy of noncommunicable diseases. J. Med. Econ..

[45] Zaman K., Ahmad A., Hamzah T., Yusoff M. (2016). Environmental Factors Affecting Health Indicators in Sub-Saharan African Countries: Health is Wealth. Soc. Indic. Res..

[46] Yang T., Liu W. (2018). Does air pollution affect public health and health inequality? Empirical evidence from China. J. Clean Prod..

[47] Lu Z., Chen H., Hao Y., Wang J., Song X., Mok T. (2017). The dynamic relationship between environmental pollution, economic development and public health: Evidence from China. J. Clean Prod..

[48] Hao Y., Liu S., Lu Z.-N., Huang J., Zhao M. (2018). The impact of environmental pollution on public health expenditure: Dynamic panel analysis based on Chinese provincial data. Environ. Sci. Pollut. Res..

[49] Khoshnevis Yazdi S., Khanalizadeh B. (2017). Air pollution, economic growth and health care expenditure. Econ. Res. Ekon. Istraz..

[50] Ghorashi N., Alavi Rad A. (2017). CO_2_ Emissions, Health Expenditures and Economic Growth in Iran: Application of Dynamic, Simultaneous Equation Models. J. Community Health Res..

[51] Chaabouni S., Zghidi N., Mbarek M.B. (2016). On the causal dynamics between CO_2_ emissions, health expenditures and economic growth. Sustain. Cities Soc..

[52] Ghosh S. (2010). Examining carbon emissions economic growth nexus for India: A multivariate cointegration approach. Energy Policy.

[53] Hartwig J. (2010). Baumol’s Diseases: The Case of Switzerland. Swiss Soc. Econ. Stat..

[54] Wang Z., Asghar M., Zaidi S., Wang B. (2019). Dynamic linkages among CO_2_ emissions, health expenditures, and economic growth: Empirical evidence from Pakistan. Environ. Sci. Pollut. Res..

[55] Romm J., Ervin C. (1996). How Energy Policies Affect Public Health. Public Health Rep..

[56] Erickson L., Jennings M. (2017). Energy, Transportation, Air Quality, Climate Change, Health Nexus: Sustainable Energy is good for Our Health. AIMS Public Health.

[57] Remoundou K., Koundouri P. (2009). Environmental Effects on Public Health: An Economic Perspective. Int. J. Environ. Res. Public Health.

[58] Jebli M. (2016). On the causal links between health indicator, output, combustible renewables and waste consumption, rail transport, and CO_2_ emissions: The case of Tunisia. Environ. Sci. Pollut. Res..

[59] Çetin M. The Long Run Relationship between Health Expenditure and Renewable Energy Consumption in BRICS-T Countries: Panel ARDL Evidence. Proceedings of the 5th International Congress on Political, Economic and Social Studies (ICPESS).

[60] Khan S. (2019). The Role of Renewable Energy, Public Health Expenditure, Logistics and Environmental Performance in Economic Growth: An Evidence from Structural Equation Modelling. Preprints.

[61] Pruss-Ustun A., Bartram J., Clasen T., Colford J.M., Cumming O., Curtis V., Bonjour S., Dangour A.D., De France J., Freeman M.C. (2014). Burden of disease from inadequate water, sanitation and hygiene in low- and middle-income settings: A retrospective analysis of data from 145 countries. Trop. Med. Int. Health.

[62] Eurostat Causes of Death (hlth_cdeath). https://ec.europa.eu/eurostat/cache/metadata/en/hlth_cdeath_esms.htm#stat_pres1562768006303.

[63] World Bank DataBank. World Development Indicators. https://databank.worldbank.org/indicator/NY.GDP.MKTP.KD.ZG/1ff4a498/Popular-Indicators.

[64] Eurostat Database, 2019. https://ec.europa.eu/eurostat/data/database.

[65] Breusch T., Pagan A. (1980). The Lagrange multiplier test and its applications to model specification in econometrics. Rev. Econ. Stud..

[66] Pesaran M. (2004). General diagnostic tests for cross section dependence in panels. CESIFO Working Paper Series No. 1229.

[67] Im K., Pesaran H., Shin Y. (2003). Testing for Unit Roots in Heterogeneous Panels. J. Econom..

[68] Choi I. (2001). Unit Root Test for Panel Data. J. Int. Money Financ..

[69] Maddala G., Wu S. (1999). A Comparative Study of Unit Root Tests with Panel Data and A New Simple Test. Oxf. B Econ. Stat..

[70] Pesaran M., Smith R. (1995). The role of theory in econometrics. J. Econom..

[71] Pesaran M., Shin Y., Smith R. (1999). Pooled Mean Group Estimation of Dynamic Heterogeneous Panels. J. Am. Stat. Assoc..

[72] Pesaran M., Shin Y., Smith R. (2001). Bounds testing approaches to the analysis of level relationships. J. Appl. Econ..

[73] Pedroni P. (1999). Critical values for cointegration tests in heterogenous panels with multiple regressors. Oxf. B Econ. Stat..

[74] Gerdtham U., Sogaard J., Andersson F., Jonsson B. (1992). An econometric analysis of health care expenditure: A cross-section study of the OECD countries. J. Health Econ..

[75] Wickens M.R., Breusch T.S. (1998). Dynamic specification, the long run and the estimation of transformed regression models. Econ. J..

[76] Kiymaz H., Akbulut Y., Demir A. (2006). Tests of stationarity and cointegration of health care expenditure and gross domestic product: An application to Turkey. Eur. J. Health Econ..

[77] Zheng X., Yu Y., Zhang L., Zhang Y. (2010). Does Pollution Drive up Public Health Expenditure? A Panel Unit Root and Cointegration Analysis. http://www.hanqing.ruc.edu.cn/admin/uploadfile/201005/20100520103320946.pdf.

[78] Bungau S., Tit D.M., Fodor K., Cioca G., Agop M., Iovan C., Cseppento D.C.N., Bumbu A., Bustea C. (2018). Aspects Regarding the Pharmaceutical Waste Management in Romania. Sustainability.

[79] Jurca T., Marian E., Vicaş L.G., Mureşan M.E., Fritea L., Sharmin E., Zafar F. (2017). Metal Complexes of Pharmaceutical Substances. Spectroscopic Analyses-Developments and Applications.

[80] Usman M., Ma Z., Wasif Zafar M., Haseeb A., Ashraf R.U. (2019). Are Air Pollution, Economic and Non-Economic Factors Associated with Per Capita Health Expenditures? Evidence from Emerging Economies. Int. J. Environ. Res. Public Health.

[81] Hargens L. (2008). Product-Variable Models of Interaction Effects and Causal Mechanisms. Working Paper No. 67R.

[82] Dumitrescu E.-I., Hurlin C. (2012). Testing for Granger non-causality in heterogeneous panels. Econ. Model..

[83] Lee J.-A., Park J., Kim M. (2015). Social and Physical Environments and Self-Rated Health in Urban and Rural Communities in Korea. Int. J. Environ. Res. Public Health.

